# Elimination of Endogenous Toxin, Creatinine from Blood Plasma Depends on Albumin Conformation: Site Specific Uremic Toxicity & Impaired Drug Binding

**DOI:** 10.1371/journal.pone.0017230

**Published:** 2011-02-28

**Authors:** Ankita Varshney, Mohd Rehan, Naidu Subbarao, Gulam Rabbani, Rizwan Hasan Khan

**Affiliations:** 1 Interdisciplinary Biotechnology Unit, Aligarh Muslim University, Aligarh, India; 2 School of Information Technology, Centre for Computational Biology and Bioinformatics, Jawaharlal Nehru University, New Delhi, India; Massachusetts Institute of Technology, United States of America

## Abstract

Uremic syndrome results from malfunctioning of various organ systems due to the retention of uremic toxins which, under normal conditions, would be excreted into the urine and/or metabolized by the kidneys. The aim of this study was to elucidate the mechanisms underlying the renal elimination of uremic toxin creatinine that accumulate in chronic renal failure. Quantitative investigation of the plausible correlations was performed by spectroscopy, calorimetry, molecular docking and accessibility of surface area. Alkalinization of normal plasma from pH 7.0 to 9.0 modifies the distribution of toxin in the body and therefore may affect both the accumulation and the rate of toxin elimination. The ligand loading of HSA with uremic toxin predicts several key side chain interactions of site I that presumably have the potential to impact the specificity and impaired drug binding. These findings provide useful information for elucidating the complicated mechanism of toxin disposition in renal disease state.

## Introduction

The uremic syndrome is attributed to the progressive retention of a large number of biologically/biochemically active endogenous solutes called “uremic toxins”, which under normal conditions are excreted by the healthy kidneys [1], [2]. The accumulation of these human metabolic products in blood has been implicated in a number of toxic effects in uremic patients, including cardio-vascular damage, progressive loss of glomerular filtration, bleeding tendencies from platelet dysfunction, hypertension, neuropathy, irregularities in thyroid function, and defective protein binding of medicinal preparations [3]–[5].

Among the highly increased uremic guanidino compounds (GCs), creatinine (CTN; 2-Amino-1-methyl-5H-imidazol-4-one, C_4_H_7_N_3_O), is the most typical example of small water-soluble breakdown waste product generated from muscle metabolism which can easily be removed by any dialysis strategy [6], [7]. CTN have been used as a conventional biomarker that predicts several important health outcomes, to diagnose acute kidney injury involving the measurement of levels of serum creatinine, blood urea nitrogen, and urinary enzymes, all of which are elevated after substantial kidney function is lost [8]. Its concentration for a healthy person is in the range of 35–106 µM whereas for a person with uremia reaches upto µM [6]. The normal level of albumin-to-creatinine ratio (ACR) in blood depends highly on sex, body muscle mass, age, racial/ethnic groups [9] and diseased states. ACR predicts several health outcomes such as neurotoxicity [10], hypertension [11], [12], and vascular damage due to leukocyte activation [13], kidney failure [14], cardiovascular events, [15] and microalbuminuria [16]. In renal diseases, the pharmacokinetics of many drugs are altered even when the primary route of elimination is not renal, due to changes in protein binding, volume of distribution, and/or acid-base disturbance [17].

We elucidate the interaction of CTN during the pH dependent structural transition, often referred to as the Neutral (N) – Basic (B) transition [18], [19] of Human serum albumin (HSA) which regulates the volume of circulating plasma; therefore, albumin must be conserved by the body [20]. The structural transitions and toxin binding properties were evaluated by means of calorimetric and, spectroscopic approaches using typical site-specific bound drugs (warfarin, phenylbutazone, ibuprofen and diazepam). The competitive binding of a toxin affects the transport of endogenous as well as exogenous substances especially site specific drugs targeted to the focus of disease for therapeutic effect [20]. The elimination of such toxins from the blood stream of patients suffering from chronic renal failure which was an important therapeutic goal was found to be dependent on albumin conformation. We determine the high affinity binding site of CTN on HSA using displacement, molecular docking and surface accessibility. The major amino acid residue being involved in the interaction was Arg257 which provides the guanidino group to the toxin. The primary binding site of toxin was located in the vicinity of Arg257 or near to loop 4 and 6 in subdomain IIA which corresponds approximately to amino acid position of 190–300, one of the two principal sites on HSA for small ligands [20]. Thus, binding of CTN to its high affinity (site I) and low affinity (site II) sites indirectly displaces drugs from albumin and increases the transiently liberated toxin molecules leading to impaired drug binding.

## Materials and Methods

### Materials

Human serum albumin (lot No. A1887; essentially fatty acid free and globulin), creatinine (lot No. C4255), warfarin (lot No. A2250), ibuprofen (lot No. I4883) and phenylbutazone (lot No. P8386) were purchased from Sigma. Diazepam was a product from Ranbaxy Laboratories Ltd., India. All of the other reagents were of analytical grade.

### Preparation of HSA Isomers and creatinine solutions

The protein and toxin solutions were prepared in pH 7.0 (60 mM sodium phosphate) and pH 9.0 (10 mM glycine-NaOH) buffer solutions. The protein concentration used was similar to that of albumin concentration present in blood i.e. 500 µM and was determined spectrophotometrically using 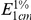 of 5.31 [21] at 279 nm on a Hitachi spectrophotometer, model U-1500. The concentration of creatinine (M_w_ = 113.12) used varies accordingly from normal serum creatinine (106 µM) to uremic conditions (2000 µM) as previously analyzed in a survey of patients suffering from uremic disorders [6]. For all measurements we have used three form of protein preparations as ‘free HSA’ not complexed with toxin; ‘normal condition’ describes the solution of HSA complexed with minimal craetinine concentration and ‘uHSA’ describes the term used for the protein/toxin complex responsible for maximal uremic conditions. In all of these preparations, we have used HSA/CTN ratio similar to that of in-vivo conditions i.e. ACR = 5 (normal condition) and ACR = 0.25 (maximal uremic condition).

### Differential Scanning Calorimetry (DSC)

The thermal denaturation of the proteins was carried out on a VP-DSC microcalorimeter (MicroCal Inc., Northampton, MA). The thermograms were obtained in the temperature range of 30–90°C at a scanning rate of 0.5 K/min. Before being loaded into the cells, the sample (HSA or HSA-CTN) and the reference (buffer or buffer-CTN) solutions were degassed by stirring in an evacuated chamber at room temperature. The solution with the vial was weighed before and after degassing, and the appropriate amount of degassed deionized water was added to make up for any loss of water thus evaporated. The solutions were then immediately loaded into the respective cells. The calorimetric reversibility of the thermal transitions was determined by heating the sample to a temperature that was a little over the transition maximum, cooling immediately, and then reheating. The reversible non-two-state denaturation model provides the temperature where the area under the transition curve is half complete (ΔT_m_), van't Hoff (ΔH_VH_) and calorimetric (ΔH_m_) enthalpies depicting actual heat absorption during protein unfolding. The cooperative unit is defined by the ratio ΔH_m_/ΔH_vH_ [22].

### Isothermal Titration Calorimetry (ITC)

The energetic of the binding of creatinine to HSA were measured using a VP-ITC titration microcalorimeter (MicroCal Inc., Northampton, MA). All the solutions were thoroughly degassed before loading, and the consequent water loss was compensated using degassed deionized water. The reactant (500 µM protein solution) was placed in the sample cell (1.4 ml) and the injectant (2000 µM creatinine solution) was introduced into the calorimeter in 8 µl increments spaced 400 sec apart. The injection syringe rotated at a speed of 300 rpm throughout the experiment to facilitate mixing of the reaction components. Sequential titrations were performed to ensure full occupancy of the binding sites by loading and titrating with the same ligand without removing the samples from the cell until the titration signal was essentially constant. To correct for the dilution effect by the injection of toxin solution, two controls were obtained: titration of HSA solution by the buffer to account for HSA dilution and titration of buffer solution by creatinine solution to account for toxin dilution effect. Experiments were repeated two or more times to get a reproducibility of better than 3%. The generated were integrated using the single set of identical binding sites model of Origin 7 software provided by MicroCal. The experimental data were best fitted to a binding model depending upon the least chi-square values obtained. The enthalpy change for each injection was calculated by integrating the area under the peaks of the recorded time course of change of power and then subtracted with the control titrations. The other thermodynamic parameters were calculated according to the formulas [23], [24]:
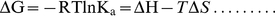
(i)Where T is the absolute temperature (298K) and R = 8.3151 J mol^−1^ K^−1^.

### Fluorescence Measurements

The intrinsic fluorescence properties of the protein were studied on a Hitachi spectrophotometer, model F-4500. The fluorescence spectra were measured at 25±0.1°C with a 1 cm path length cell. The excitation and emission slits were set at 10 and 20 nm, respectively. Intrinsic fluorescence was measured by exciting the protein solution at 280 and 295 nm to selectively excite the chromophoric molecules, and the emission spectra were monitored in the wavelength range 300–500 nm. The emission spectra of the protein-toxin solutions were subtracted from the buffer-toxin blanks, and an average of three accumulated scans was recorded as the final graph. The fluorescence data were analyzed according to linear and modified stern-volmer equations as [21], [25]:
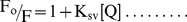
(ii)

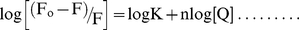
(iii)


### FT-IR Spectroscopic Measurements

Infrared spectra were recorded on a Nicolet Magna 750 FT-IR spectrophotometer (DTGS detector, Ni-chrome source and KBr beamsplitter) with a total of 100 scans and resolution of 16 cm^−1^, using AgBr windows at room temperature. The concentration of HSA was 500 µM. The difference spectra [(protein solution) - (protein solution + ligand solution)] were collected after 1 h of incubation of HSA. The protein FT-IR spectra were processed as the procedures as reported by Kang et al. [26].

### Determination of the Protein Secondary Structure

The secondary structure content of HSA and the HSA complexed with toxin under normal and maximal uremic conditions was determined from the shape of the amide I band, located around 1650–1660 cm^−1^. The FT-IR spectra were smoothed, and their baselines were corrected automatically using the built-in software of the spectrophotometer (OMNIC version 3.1). Each Lorentzian band was assigned to a secondary structure according to the frequency of its maximum; α-helix (1656–1658 cm^−1^), β-sheet (1614–1638 cm^−1^), turn (1660–1677 cm^−1^), random coil (1640–1648 cm^−1^), and β-antiparallel (1680–1692 cm^−1^) were adjusted and the area was measured with the Gaussian function. The relative percentage of the secondary structural elements was obtained from the area under the Gaussian curve [27].

### CD and UV spectroscopic Measurements

CD and UV spectra were recorded on a Jasco J-815 spectropolarimeter, equipped at 25±0.2°C in a rectangular cuvette with 1 cm pathlength under a constant nitrogen flow. Each spectrum was signal-averaged at least two times with a bandwidth of 1.0 nm and resolution of 0.2 nm, at a scan speed of 20 nm/min. Temperature control was provided by a Peltier thermostat equipped with magnetic stirring. Stock solutions of the site specific markers 6.8×10^3^ µM warfarin, 6.8×10^3^ µM phenylbutazone, 1.2×10^4^ µM ibuprofen, 8×10^3^ µM diazepam were prepared and added stepwise in µl volumes to the creatinine–HSA solutions both at pH 7.0 and 9.0. These solutions were prepared by dissolving in ethanol such as its concentration never exceeded 13% and the effects of the organic solvent on the CD measurements were undetectable. Induced CD spectra resulting from the interaction of the toxin with HSA were obtained by subtracting the CD spectrum of the protein from that of the protein-toxin complex. Ellipticities values were converted to ‘Δε’ values using the equation Δε = θ/(33982cl) where, Δε is the molar circular dichroic absorption coefficient expressed in M^−1^cm^−1^, c is the concentration of the sample expressed in mol/L, and l is the pathlength through the cell expressed in cm.

### Molecular Docking Studies

The crystal structure of HSA site I markers (warfarin, PDB ID: 1H9Z; phenylbutazone, PDB ID: 2BXC) and the site II markers (diazepam, PDB ID: 2BXF; ibuprofen, PDB ID: 2BXG) were derived from Protein Data Bank. The residues falling within 5 Å distance of the marker were extracted and combined to define the binding site. The two dimensional (2D) structures of ligands were extracted from Pubchem database in SDF (Structure Data File) format. The three dimensional structures were generated with CORINA version 2.6 [28]. Molecular docking simulations of uremic toxin and site specific probes were carried out using the GOLD version 3.1.4 program (Genetic Optimization Ligand Docking) [29] software which uses a genetic algorithm to calculate the possible conformations of the toxin that binds to the protein. The ligands were docked to active site of HSA using standard set parameters of GOLD throughout the simulations. For each of the 100 independent genetic algorithm runs, a selection pressure of operations were set to terminate after a maximum of 2,500,000 energy evaluations. Lowest energy complex geometries and the corresponding free energy of binding were calculated. Top 20 poses were saved for each ligand and best score values were used to correlate with experimental data. The binding energies of docked molecules were also calculated using X-score [30]. The hydrogen bonding (cutoff distance of 2.8–3.2 Å between donor and acceptor) and hydrophobic interactions between ligand and protein were calculated using Getneares, a program available with DOCK version 3.1.4 [31]. PyMol version 0.99 [32] and chimera version 1.3 [33] were used for visualization and measurement of distances between the ligand and the receptor.

The Accessible Surface Area (ASA) of uncomplexed HSA and complexes of ligands with HSA were calculated using NACCESS version 2.1.1 [34]. The structure of the ligands corresponding to the final docked conformation was chosen and composite coordinates were generated to form the docked complex. The change in ASA (ΔASA) of the i^th^ residue was calculated using the expression: 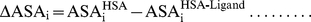
(iv)If a residue lost more than 10 Å^2^ ASA on going from the uncomplexed to the complexed state, it was considered as being involved in interaction [35].

## Results

### Thermal Denaturation of N and B isomer of HSA in presence of creatinine

**Figure 1:**
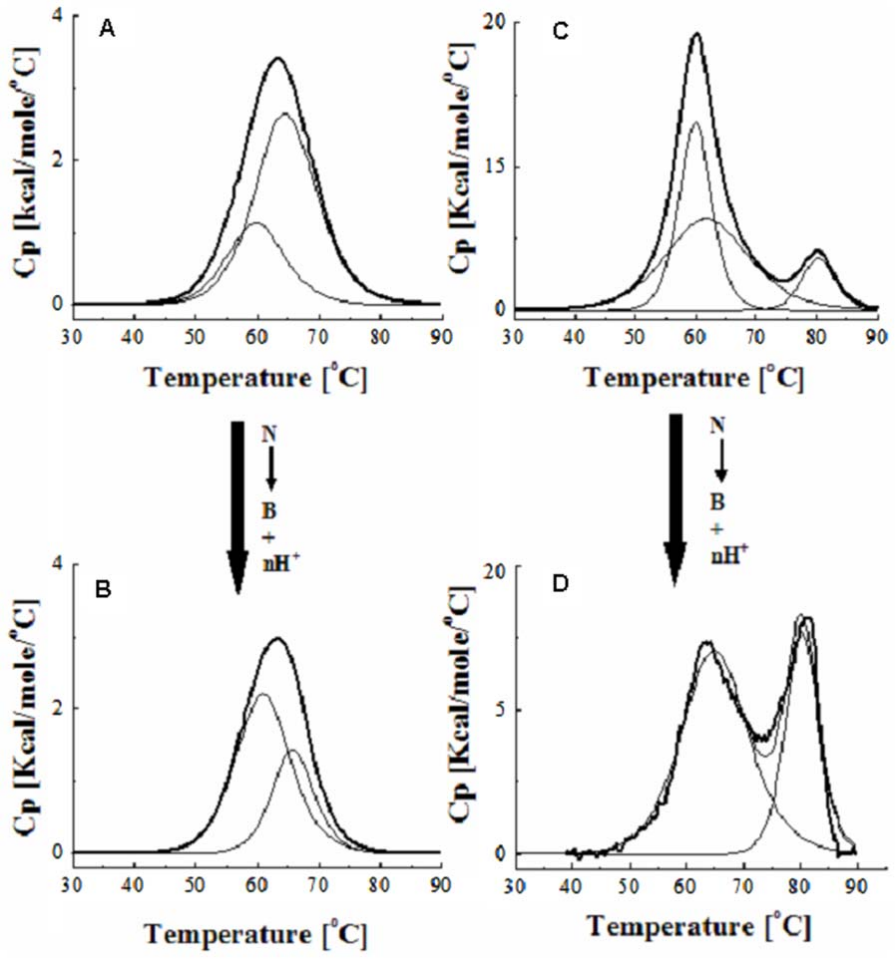
Differential scanning calorimetry of HSA in absence and presence of creatinine. Melting thermograms of Human Serum Albumin under normal (A and B) and uremic (C and D) conditions at pH 7.0 (A and C) and pH 9.0 (B and D).

The representative differential scanning calorimetric profiles of thermal denaturation of N and B isomers of HSA in absence and presence of creatinine were performed under normal and uremic condition as shown in Figure 1 (for simplicity we have only shown data for native protein at pH 7.0 (A) pH 9.0 (B) and maximal uremic conditions at pH 7.0 (C) and 9.0 (D) respectively. The corresponding thermodynamic parameters accompanying the transitions were reported in Table 1. In the absence of toxin, N isomer of HSA unfolds with reversible endotherm at transition temperatures of 59–64°C and an average calorimetric enthalpy of 14.2–35.2 kcal/mol. Almost similar value of the transition temperature of N and B isomeric forms (in absence of toxin) indicates the extent of the binding interactions of positive charge of amino acid residues participating in the molecular mechanism of this equilibrium. However, a little difference in the cooperativity ratio β was found to be 1.02±0.01 (data not shown), indicating that the thermal unfolding of the protein in the presence of toxin even at higher concentrations was a reversible process. These values were in concurrence with those reported in the literature [36]. The melting curve of uremic plasma (albumin complexed with creatinine) shows a different shape without any reversal in amplitude of the two peaks but a slight shift to the right at higher temperature. The toxin renders the strongest influence on the endotherms (more pronounce for basic isomer of HSA, Figure 1D) by transforming the unimodal curve of its melting to a bimodal one. As it follows from Figure 1, on reaching from normal to maximal uremic condition, the melting curve was practically identical to that for the complex “albumin-nonconjugated bilirubin” [37].

**Table 1:** Thermodynamic parameters accompanying thermal unfolding of HSA complexes with creatinine at a scan rate of 0.5 K/min.

Condition	T_m_ [K]		ΔH_m_ [Kcal/mol]	
	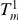 a	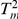 a	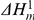 b	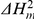 b
**A**	59.84±0.63	64.59±0.28	14.2±0.52	35.2±0.54
**B**	61.01±0.51	65.83±0.35	26.2±0.38	13.2±0.38
**C**	59.93±0.02	80.27±0.05	143.00±0.52	39.31±0.09
**D**	65.06±0.12	80.31±0.07	110.30±0.96	58.69±0.15

Condition A: N Isomer of HSA at pH 7.0.

Condition B: B Isomer of HSA at pH 9.0.

Condition C: N Isomer of HSA at pH 7.0+ CTN.

Condition D: B Isomer of HSA at pH 9.0+ CTN.

a Midpoint of thermal denaturation.

b Calorimetric enthalpy.

Superscripts 1 and 2 refer to the low and high unfolding transition, respectively (see text for details).

### Isothermal Titration Calorimetry of N and B isomer of HSA in presence of creatinine

**Figure 2:**
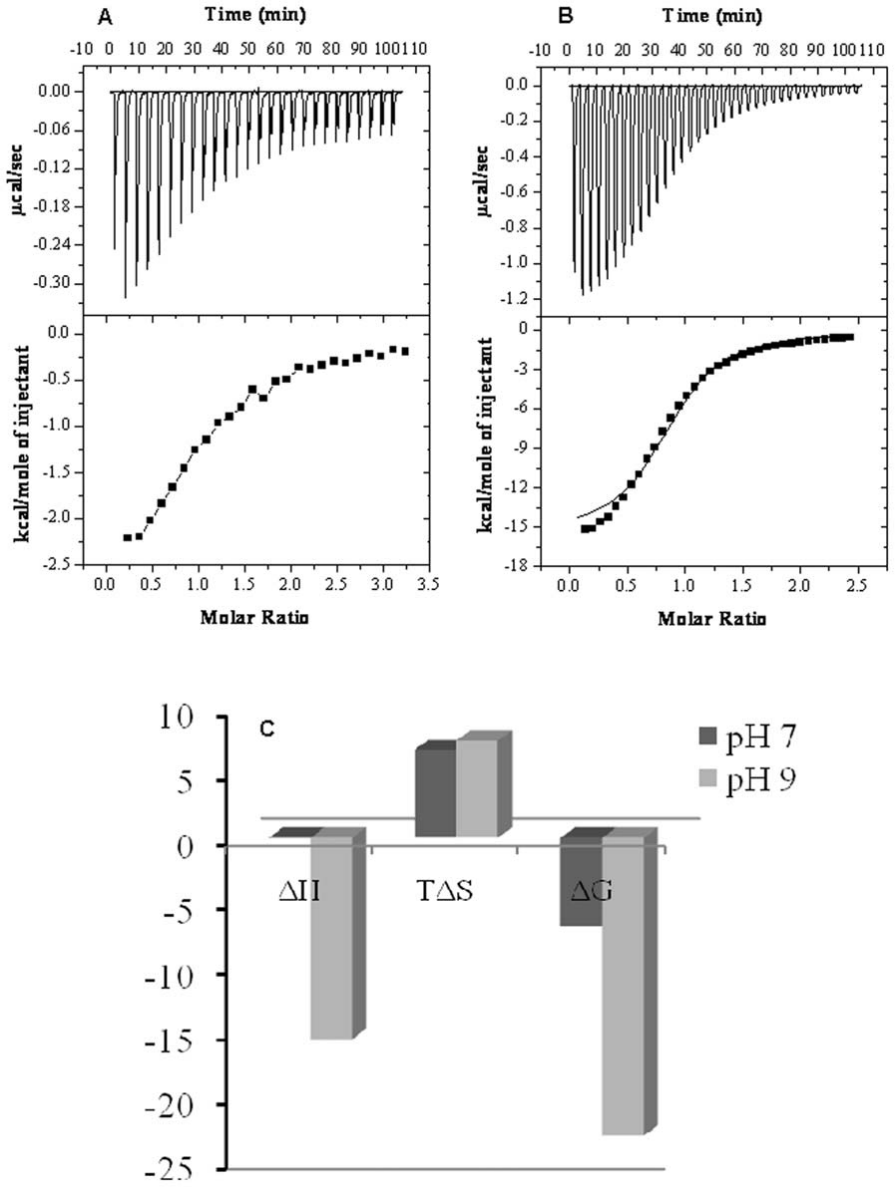
Isothermal titration calorimetry of HSA complexed with creatinine. The ITC experiments of N and B isomers of Human serum albumin at pH 7.0 (A) and 9.0(B) respectively were performed at 25°C. In the top panels, the heat released per unit time (µcal/sec) was plotted vs time where each peak corresponds to the injection of an aliquot of ligand. In the bottom panels, the heat of reaction per injection (kcal/mole) was determined by integration of the area under each peak, plotted vs [CTN]/[HSA], and fit using the software provided by Microcal. (C) Comparative distribution of ΔH, TΔS and ΔG at pH 7.0 (dark grey) and pH 9.0 (light grey).

In order to determine thermodynamic parameters for binding we performed isothermal titration calorimetry of creatinine with neutral and basic isomer of HSA. A representative calorimetric titration profile of ACR at molar ratio of 1∶4 (HSA in normal serum: HSA complexed with creatinine under uremic conditions) at pH 7.0 (A) and pH 9.0 (B) were shown in Figure 2. Each peak in the binding isotherms (Figure 2, upper panels) represents a single injection of creatinine. The negative deflections from the baseline on addition of creatinine indicate that heat was evolved (an exothermic process). The enthalpy change associated with each injection of ligand was plotted versus the [CTN]/[HSA] molar ratio (Figure 2, lower panel), and the ΔH, K_a_, the free energy change (ΔG) associated with binding were determined from the plots. The thermodynamic data derived from the model fitting were summarized in Table 1. The 1∶4 binding stoichiometry of CTN to basic isomer of HSA with a binding constant of 2.69×10^5^ M^−1^ indicates a strong and specific interaction. This binding constant was of larger order of magnitude than that of neutral form (8.92×10^4^ M^−1^) as shown in Table 2. Furthermore, the heat released during the CTN-HSA reaction increases with increasing pH, i.e., the ion pair attraction and H-bonds between CTN and HSA were weakened. This indicates that the number of H-bonds formed by CTN was lower at pH 7.0. In contrast, ΔS become more negative with increasing pH. This indicates that CTN binding destroyed the internal hydrophobic interactions in HSA at pH 9.0, replacing them with ion pair attraction and H-bonds. Comparison of the ΔG, ΔH, and TΔS values suggests that the CTN-HSA interaction was amphipathic and H-bonds and ion pair binding were both major contributors, i.e., the interaction of CTN with HSA depends on a combination of ion pair attraction and H-bonds (Figure 2C).

**Table 2:** Association constants and thermodynamic data for binding of creatinine to HSA.

Condition	K_a_[M^−1^]		n		ΔG^a^[Kcal/mol]	ΔH^a^[Kcal/mol]	ΔS^a^[cal/mol/K]
	ITC^a^	Spec^b^	ITC^a^	Spec^b^			
**A**	8.92×10^4^	^*^1.15×10^4^^#^2.92×10^4^	0.98	^*^0.94^#^1.07	−6.75	−0.017±0.91	22.6
**B**	2.69×10^5^	^*^9.76×10^5^^#^1.36×10^5^	0.94	^*^1.29^#^1.17	−23.03	−15.53±0.79	−25.2

Condition A: N Isomer of HSA at pH 7.0+ CTN.

Condition B: B Isomer of HSA at pH 9.0+ CTN.

a Constants determined by Isothermal Titration Calorimetry.

b Constants determined by Fluorescence spectroscopy (^*^λ_excitation_ = 280 nm; ^#^λ_excitation_ = 295 nm).

### Analysis of secondary structure of N and B isomer of HSA in presence of creatinine

**Figure 3:**
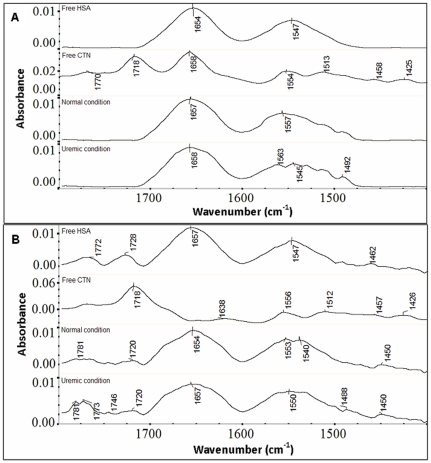
Fourier transform infrared (FTIR) measurements of HSA in absence and presence of creatinine. Infrared spectra were recorded on a FTIR spectrometer in the region 1800–1400 cm^−1^ at pH 7.0 (A) and pH 9.0 (B) for the free HSA, free CTN and difference spectra of HSA-CTN complexes (bottom two curves) obtained under normal serum (ACR = 5) to uremic condition (ACR = 0.25) (indicated in the figure).

**Figure 4:**
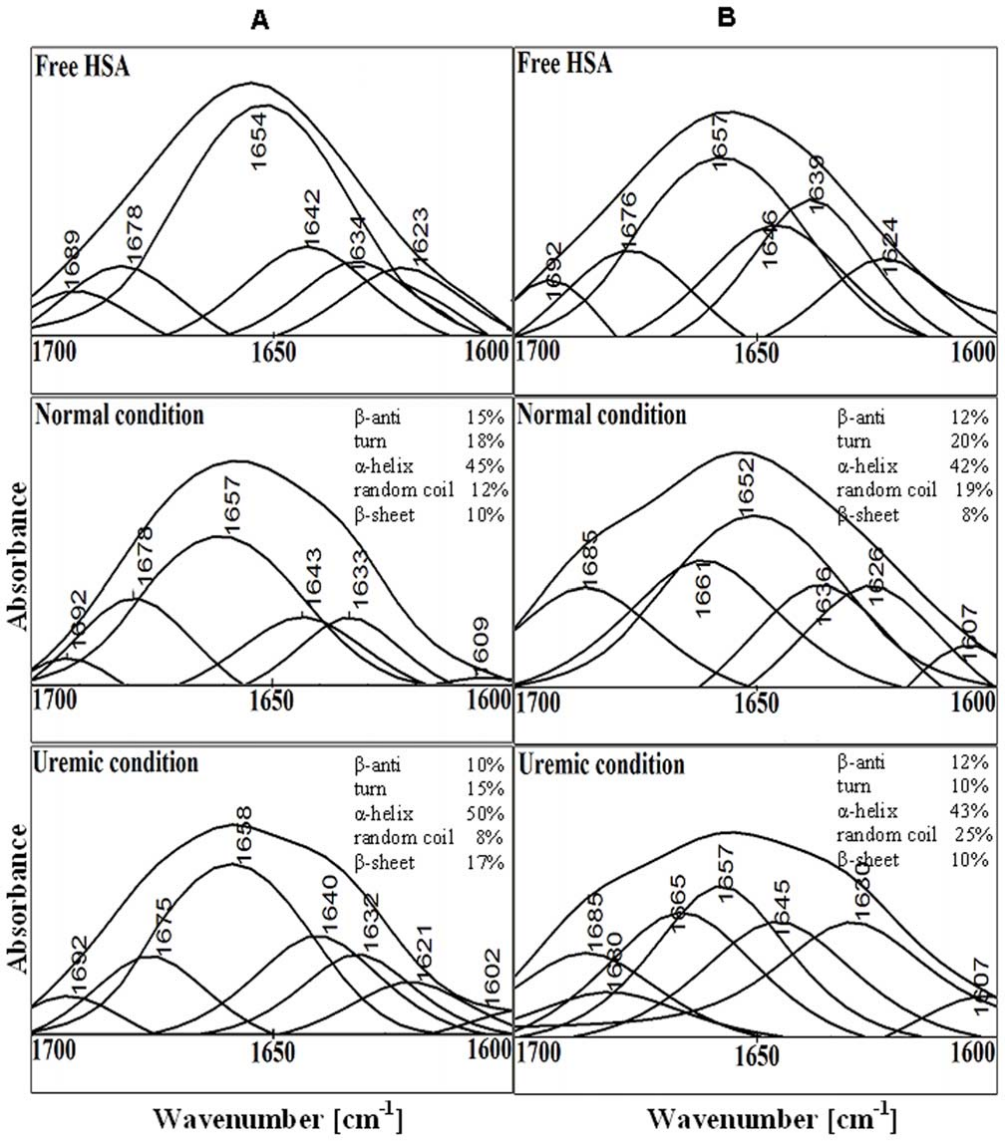
Determination of protein secondary structure complexed with creatinine. Curve fitted amide I region (1700–1600 cm^−1^) with secondary structure determination of the free HSA and its toxin adducts in aqueous solution with varying ACR molar ratios and 500 µM protein concentrations at pH 7.0 (A) and pH 9.0 (B).

To investigate the effects of the uremic toxin creatinine on the secondary structure of albumin, we analyzed regions of IR spectra caused by vibrations of polypeptide backbone, viz., the amide I (1700–1600 cm^−1^, mainly C = O stretch) band and the amide II (1500–1600 cm^−1^, C-N stretching coupled with N-H bending modes) band. The amide I band of free HSA (Figure 3) had a major maximum around 1654 cm^−1^ for native and 1657 cm^−1^ for basic isomer, characteristic of the α-helical conformation [34]. Similarly, the infrared self-deconvulation and curve fitting procedures were used to determine the protein secondary structure under normal and uremic conditions both at pH 7.0 and 9.0 (Figure 4, Table 3).

**Table 3:** FT-IR/ATR determination of secondary structure percentages of N and B isomers of HSA and its uremic complexes.

Amide I components	N isomer			B isomer		
	Free HSA(%)	Normal^a^(%)	Uremic^b^(%)	Free HSA(%)	Normal^a^(%)	Uremic^b^(%)
**β-antiparallel**(1675–1695 cm^−1^)	8±2	15±1	10±1	10±2	12±2	12±1
**Turns**(1666–1673 cm^−1^)	15±1	18±1	15±1	15±2	20±1	10±2
**α-helix**(1650–1658 cm^−1^)	54±2	45±1	50±2	53±1	42±2	43±2
**Random coil**(1637–1645 cm^−1^)	6±1	12±1	8±1	15±2	19±2	25±1
**β-sheets**(1613–1625 cm^−1^)	17±1	10±1	17±1	7±2	8±2	10±2

a Normal condition: HSA complexed with creatinine under normal serum condition.

b Uremic condition: HSA complexed with creatinine under maximal uremic condition.

These complexes were obtained incubating uremic toxin with 500 µM of HSA for 1 hr at room temperature. Data represent average obtained from two independent replicates, standard error is indicated.

Positions and relative intensities of the components in presence of toxin (i.e. uremic condition) did not differ significantly from those of normal HSA (free HSA without toxin). Though quantitative analysis of the amide I (Table 3) revealed a substantial decrease in the amount of α-helical conformation and an increase in β-sheets and or/extended chains in uremic HSA. The decrease in the intensity of amide I and amide II bands, mainly C = O and C-N vibrations, compared to low toxin concentration (normal condition) suggests major protein conformational changes upon HSA-toxin interaction possibly caused by a reorganization of intra- and intermolecular hydrogen bonding. The toxin-HSA complexation suggests partial protein unfolding more pronounced at higher molar ratio (uremic condition) i.e. similar to uremic diseased state and for basic isomeric form of HSA. The IR spectra of uremic HSA (HSA complexed with toxin) at higher molar ratios for both isomers showed appearance of some new components for amide II band (Figure 3). This band was caused by vibrations of the peptide N-H groups and by motions of Glu, Asp, Tyr, Lys and His side chains. We observed main alterations in the range 1652–1695 cm^−1^ that were basically associated with that of Arg environment. Because the appearance of this component in the IR spectra of uHSA was accompanied by no changes in the peptide C = O absorption, it likely reflects alterations in the environment of Glu and Asp side chains in uHSA molecules [38].

### Fluorescence spectroscopy of N and B isomer of HSA in presence of creatinine

**Figure 5:**
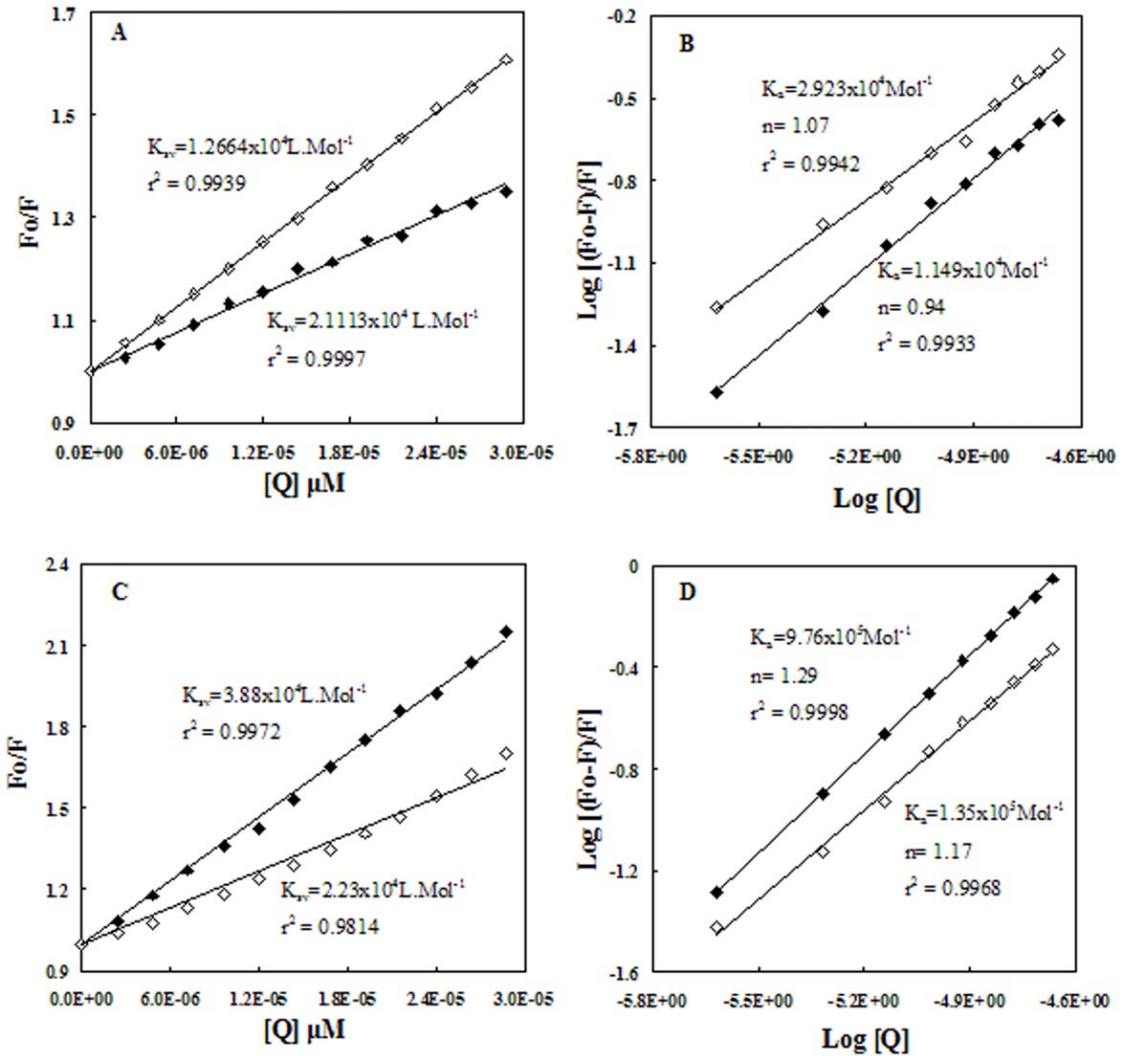
Fluorescence quenching of HSA with creatinine at different ligand/protein ratios. Stern-Volmer (A&C) and modified Stern-Volmer (B&D) plots of N (A&B) and B (C&D) isomeric conformations of HSA with uremic toxin creatinine. Each data point was the mean of 3 independent observations (S.D. ranging 0.03–0.4%). The Protein was excited at 280 (**♦**) and 295 nm (**◊**).

Both the intensity and the position of the fluorescence emission spectrum of tryptophan were sensitive to changes in the fluorescence environment and consequently to the protein conformation. Hence, to understand the influence of creatinine binding on the neutral and basic form of HSA we studied the changes in the intrinsic fluorescence of the protein. Figure 5 shows the fluorescence spectra of HSA in the presence of increasing concentration of creatinine. The fluorescence quenching data were analyzed according to the Linear (Figure 5A and C) and modified Stern-Volmer equation (Figure 5B and D) [21], [35] after exciting the protein at 280 and 295 nm. We observed that, quenching of albumin fluorescence was not affecting the binding strength of tryptophan fluorescence. This may be so because binding of uremic toxin was not exactly at the site where tryptophan resides but it could be somewhere near to it as it mainly affects tyrosine fluorescence. These results are comparable with that of our ITC results and are presented in Table 2 further confirming that association constant K_a_ of uremic toxin depends on conformation of HSA underwent N-B transitions.

### Dependence on alkalization of the Optical properties of creatinine-HSA solution

**Figure 6:**
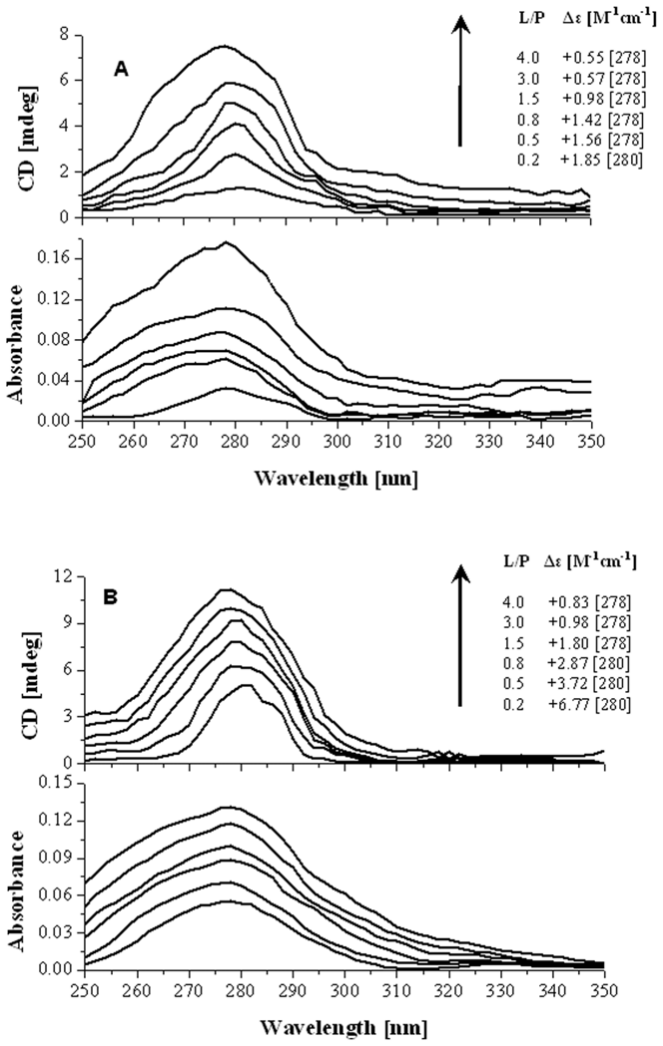
Induced circular dichroism and UV/vis spectra of creatinine-HSA complex. Representative CD and UV spectra obtained following the titration of the buffer solution of HSA with creatinine at (A) pH 7.0 and (B) pH 9.0. Spectral contributions of the protein alone were subtracted from the spectra of the toxin–protein mixture ([HSA]  = 100 µM, T = 25°C). Δε_max_ values calculated on the basis of the total ligand concentrations are displayed at the different molar ratios (L/P). Arrows denote increasing concentration of uremic toxin CTN.

The conjugated double bond system of creatinine constitutes the light absorbing chromophore that has no element of chirality; it does not show CD activity. The toxin gives the weak absorption band associated with an electronic dipole allowed Λ-Λ* transition which becomes optically active upon binding to the asymmetric environment of HSA and an induced CD spectrum emerges a weak positive Λ-Λ* CD band between 250–350 nm (Figure 6). Regarding the interaction of toxin with the albumin binding site, it has to be noted that dissociated ligand molecules lose their hydrogen donor properties but at the same time, they become more powerful proton acceptors ready to form H bonds with basic residues of HSA. Upon increasing pH value, the CD signals and absorption curves of HSA-CTN complex enhances; triggered by the protonation of histidine residues, toxin flips away at neutral and alkaline pH values and the cavity becomes accessible for the ligand molecule. The spectra obtained by toxin titration experiment (Figure 6A) clearly demonstrate that the protein microenvironment at pH 7.0 was less favourable to accommodate toxin as a chiral conformer.

### Probing the binding site of Creatinine on HSA by Ligand Displacement experiments

**Figure 7:**
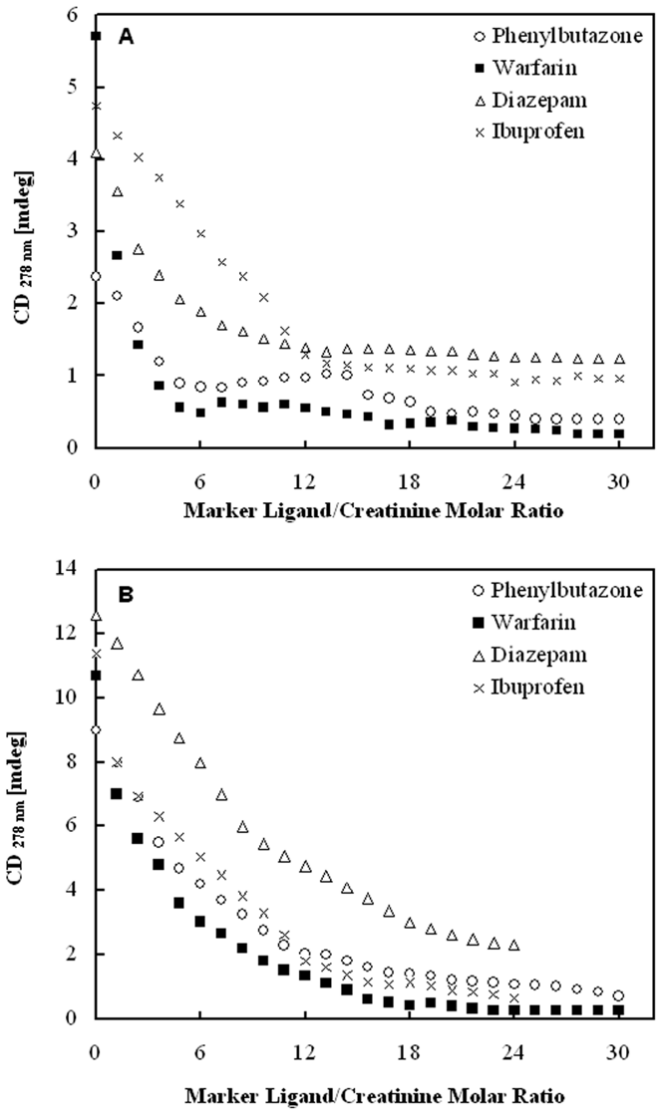
Displacing effect of HSA site specific markers on the ICD signal of creatinine-HSA complex. Results of CD displacement experiments performed with warfarin, phenylbutazone, ibuprofen and diazepam on HSA-CTN complex at (A) pH 7.0 and (B) pH 9.0 (l = 1 cm, T = 25°C). Displacers were added as µl aliquots of stock solutions. Positive induced CD values measured at 278 nm are plotted against displacer/creatinine molar ratios (for further details see Materials and methods).

Further spectroscopic experiments were undertaken to obtain information on the potential location of the HSA binding site of CTN. Albumin possesses two main drug binding sites, site I and II, which are located in hydrophobic cavities of subdomains IIA and IIIA, respectively [20]. In the presence of a compound having the same binding site as creatinine, amplitudes of the induced cotton effects should decrease due to competition. Therefore, CD displacement experiments were performed using four marker ligands warfarin and phenylbutazone for site I whereas ibuprofen and the benzodiazepine agent diazepam for site II. Monitoring the induced CD spectrum of creatinine during the titration showed a rapid extinction of extrinsic CD activity for both isomers of HSA but this extinction was found to be more steep at pH 9.0 (Figure 7). Especially the site I markers, phenylbutazone followed by warfarin were found to be responsible for reducing the CD signal of the protein-toxin complex to almost zero. Thereby, suggesting the direct competitive interaction between site I markers and creatinine for the binding site I. Notably, nearly complete extinction of the CD signal was achieved for both isomeric forms of HSA suggesting that the binding site was not affected by the protonation of albumin molecules.

### Molecular docking study of uremic toxin creatinine–HSA interaction

**Figure 8:**
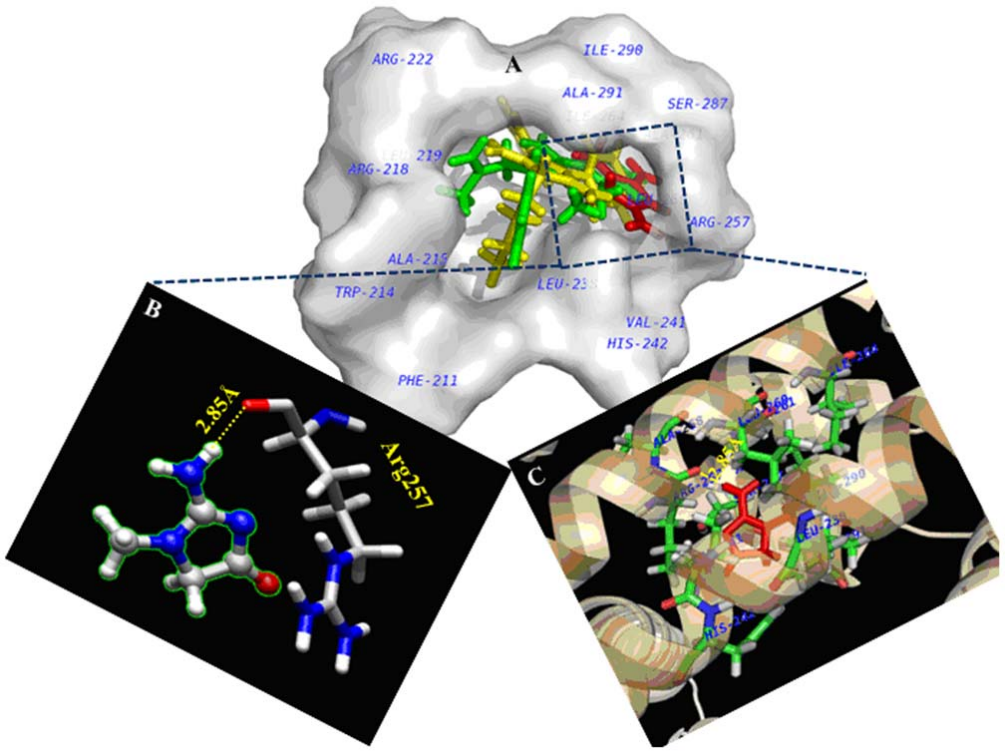
Molecular docking of HSA complexed with site I specific markers. (A) Molecular surface representations of HSA showing site I specific markers [warfarin (limegreen); phenylbutazone (yellow)] and the toxin CTN (red) at binding site I. (B) One hydrogen bond (as highlighted by the yellow dashed line) was formed between CTN and Arg257 of HSA. The hydrogen bond length was represented in yellow colour (C) Other site I amino acid residues of HSA interacting with CTN within 5 Å distance.

**Figure 9:**
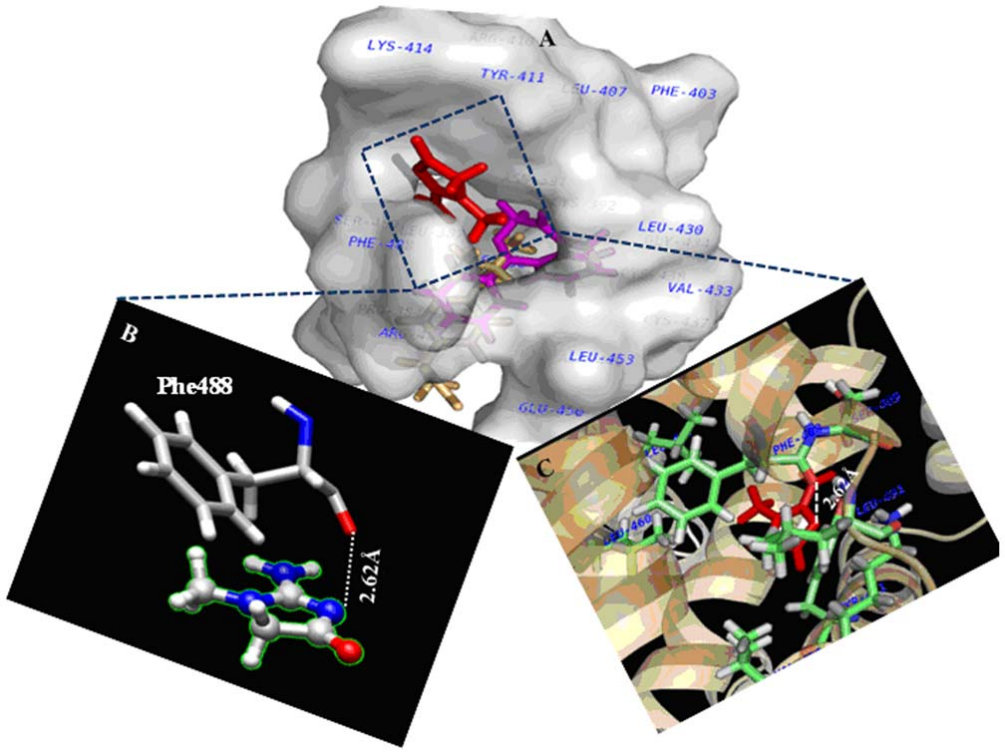
Molecular docking of HSA complexed with site II specific markers. (A) Molecular surface representations of HSA showing site II specific markers [ibuprofen (light orange); diazepam (magenta)] and the toxin CTN (red) at binding site II. (B) The toxin molecule forming repulsive interaction (white colour) with Phe488 of HSA (as highlighted by the white dashed line). (C) Other site II amino acid residues of HSA interacting with CTN within 5 Å distance.

The locations of uremic toxin creatinine in the active sites (site I and site II) of HSA was explored by conducting docking simulations using GOLD [29] as presented in Figure 8 and 9. The principal regions of ligand binding to HSA were located in hydrophobic cavities in subdomains IIA and IIIA, which are consistent with site I and site II, respectively [20]. The creatinine, site I markers (phenylbutazone and warfarin) were docked to HSA and the results have been shown in Table 4 and 5. The Gold Fitness Score, measure of binding affinity was found to be low for CTN at both of the sites when compared to respective markers used in this study; however specificity was higher for site I as evident from the presence of hydrogen bond and hydrophobic interactions. The spectroscopic experimental results were substantiated by docking results which shows that high and low affinity binding sites of toxin on plasma protein are located within the binding pocket of subdomain IIA and IIIA. The creatinine binds deep inside the cavity at site I (Figure 8) whereas in site II (Figure 9), it binds at peripheral side of the cavity. The inside wall of the pocket was lined by hydrophobic side chains whereas the entrance to the pocket was surrounded by several non-polar residues (Leu238, Val241, Ala258, Leu260, Ala261, Ile264, Ile290, Ala291); one polar (Ser287) and few charged residues (His242, Arg257) in the proximity distance of 5 Å of the bound toxin (Figure 8, Table 4). Although the involvement of non polar residues makes the interactions to be hydrophobic in nature but the strong intermolecular hydrogen bond between carbonyl oxygen atom of Arg257 and N_2_ atom of creatinine (2.85 Å, 124.36°), makes the electrostatic interaction as the primary binding force responsible for the retention of toxin in the plasma. The hydrogen bond residue of site I (Arg 257) was part of helix 4 and 6 of subdomain IIA (represented as IIa-h4 & IIa-h6, Table 4). While at site II, the complex was stabilized by hydrophobic interactions without involvement of any hydrogen bond. Additionally, electrostatic repulsive force between oxygen atom of Phe488 with N_3_ of creatinine (2.62 Å) destabilizes the complex (Figure 9, Table 5). To further identify the residues taking part in the interaction, we have calculated the accessible surface area (ASA) of the amino acid residues. The changes in ASA of the interacting residues are presented in Table 4 and 5.

**Table 4:** Change in Accessible surface area and binding interactions of the site I amino acid residues of HSA with uremic toxin creatinine.

Ligands	Amino acidresidue	ΔASA (Å^2^)	Location	Electrostatic Interaction	Number of other interacting residues (5 Å)	GOLD Fitness score	X-score
**Creatinine**	Arg257Leu238Ala291Ser287Leu260	17.9617.0816.0512.0210.58	IIa-h4IIa-h6IIa-h3IIa-h6IIa-h4	Hydrogen bondC = O…H^2^NArg257(2.85 Å)	10	28.16	−5.68
**Phenyl-butazone**	Ala 291Leu238Leu242Trp214Arg257Ile264Arg222Leu219Ser287Ile290Leu260	42.941.7933.6622.6118.9513.0412.8212.6912.0211.4811.17	IIa-h6IIa-h3IIa-h3IIa-h2IIa-h4IIa-h4IIa-h2IIa-h2IIa-h6IIa-h6IIa-h4		15	47.82	−8.49
**Warfarin**	Leu238Ala291Leu242Trp214Arg257Arg222Leu219Ser287Ile290Phe211Leu260	39.0338.635.628.9316.414.0512.2512.0211.4811.2610.72	IIa-h3IIa-h6IIa-h3IIa-h2IIa-h4IIa-h2IIa-h2IIa-h6IIa-h6IIa-h2IIa-h4		15	41.62	−8.05

**Table 5:** Change in Accessible surface area and binding interactions of the site II amino acid residues of HSA with uremic toxin creatinine.

Ligands	Amino acid residue	ΔASA (Å^2^)	Location	Electrostatic Interaction	Number of other interacting residues (5 Å)	GOLD Fitness score	X-score
**Creatinine**	Tyr 411Phe 488Glu 489	35.6626.3810.27	IIa-h2IIIa-h6IIIa-h4	Repulsive forceC = O…N^3^Phe488(2.62 Å)	7	29.14	−5.55
**Diazepam**	Leu 453Asn 391Ile 388Leu 387Leu 430Arg 485Ala 449	31.1328.2723.9318.4616.6214.6713.73	IIIa-h4IIIa-h1IIIa-h1IIIa-h1IIIa-h3IIIa-h6IIIa-h4		15	41.10	−8.43
**Ibuprofen**	Leu 453Val 485Asn 391Leu 387Ile 388Pro 384Glu 450	28.2227.5521.8219.416.8510.7910.79	IIIa-h4IIIa-h6IIIa-h1IIIa-h1IIIa-h1IIIa-h1IIIa-h4		13	42.74	−7.29

## Discussion

The HSA solutions used in our preparations was approximately equivalent to that present in the blood i.e. 500 µM. Although under this condition the HSA solution was very concentrated but we have checked for the possibility of aggregation formation while performing the experiments. No evidence was found for aggregation/turbidity in solution provided by the Rayleigh light scattering (RLS) experiments (Figure S1) in scattering intensity after 1 hr. By RLS time dependent in solutions increase was in a concentration dependent manner. It appears that no aggregation was been reported when concentration was increased from 150 µM (10 mg/ml) to higher concentrations ∼500 µM (33 mg/ml).

The melting thermograms for HSA were shown in Figure 1 attributes to an excessive load of hydrophobic uremic toxin bound to the albumin molecule. The uremic plasma under physiological conditions have a different shape with a shift to the right at higher temperatures and increase in the height above baseline which was also reflected in the values of the melting enthalpy (Table 1). This was attributed to a higher thermoresistance of the albumin component, most probably because of the binding of uremic toxin to HSA. An increase in the number of intramolecular bonds increases the thermal stability of the protein molecule and more pronounced at pH 9.0. Therefore, binding of toxin increases the cooperativity of the melting process and depends on its affinity for the albumin molecule. Comparison of thermodynamic parameters with the spectroscopic method (Table 2) shows that the two measurements yield similar ‘n’ values at both pHs. Because ΔH was much less than 60 Kcal/mol [39], the CTN-HSA interaction was non-covalent: it involves H-bonds, ion-pair attraction, hydrophobic interaction, and van der waals forces. It can be observed that, at physiological pH, the binding affinity decreases. This was reflected in the high negative values of enthalpy of binding, ΔH_ITC_, and in a larger affinity binding constant, K_ITC_ (Table 2). Our finding that the secondary structure of serum albumin from conditions similar to uremic patients remains intact was consistent with circular dichroism data on the uHSA conformation in solution as determined previously [38]. Likewise, modification of IR spectra indicating alterations in structure of uHSA agrees well with published data about the physicochemical peculiarities of albumin in chronic uremia. We do not observe any significant changes in the secondary environment of protein on complexation with the toxin. On the other hand, number of binding sites ‘n’ remained almost unaffected. This indicates increased stability of B-CTN complex. Since under increased Ca^2+^ concentration in the blood plasma, the B isomer predominates, it is suggested that N-B conformational changes have physiological significance [20], [18].

**Figure 10:**
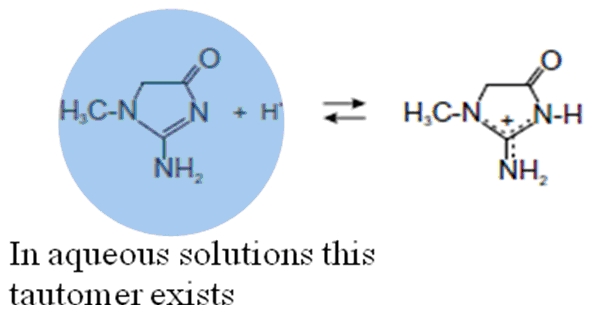
The acidic-basic equilibrium of creatinine. In aqueous solution creatinine exists in the form of a tautomer.

The question arises which binding sites on the albumin molecule were usually affected by creatinine? Data for the binding ability of albumin before and after its loading with uremic toxin with marker ligand specific for sites I and II were represented by displacement experiments as shown in Figure 7. The site I markers at pH 7 and 9 reduces the induced CD signal to almost zero. The association constant of phenylbutazone was K_a_ 7×10^5^ M^−1^, n = 1 and warfarin was K_a_ = 3.3×10^5^ M^−1^, n = 1 [20] and that for CTN (after exciting the protein at 280 nm) was K_a_ = 1.4×10^5^ M^−1^, n≈1 (Table 2) the site I markers can displace CTN from its binding site on albumin. The association constant for diazepam (K_a_ = 3.8×10^5^ M^−1^, n = 1) and ibuprofen (K_a_ = 2.7×10^5^ M^−1^, n = 1) [20] was higher than that for CTN and lower than that for site I markers. Site II markers were, therefore, likely to weak inhibitors of the binding of CTN. Thus, fluorescence quenching and induced CD spectrums were utilized to achieve three goals (i) calculating the association constant (K_a_) (ii) determining the dependence of toxin binding on albumin conformation and (iii) probing the location of uremic toxin binding site on HSA. Furthermore, CTN possesses two pKa values of 4.88 for protonation [40] and 12.7–13.4 for deprotonation of the exocyclic amino group. This suggests that CTN predominantly occurs in aqueous solution and in blood plasma at pH 7.4 in neutral form. In aqueous solution it exists in the form of a tautomer, its acido–basic equilibrium has been shown in Figure 10. When analyzing the accessible surface area we found main involvement of arginine residues in the toxin complexation with albumin (Table 4). This change seemed to be caused by His residues because most of the pK_a_ values of His residues are within this pH region i.e. 6.4. Thus, during alkalization of HSA, the affinity of CTN increases. On the onset of pH 9.0, the Arg residues involved in binding protonate (pKa of Arg  = 9.04 (NH^3+^) and 12.04 (side chain)), and then may interact favorably with the toxin. Whilst at pH 7.0, deprotonation weakens the interaction with the cation.

What actually happens under in vivo conditions during chronic renal failure (CRF) when urine eliminates from the body? The glomerulus is a selective filtration membrane. The filtration barrier of body composed of three layers that allow for the filtration of solutes (eg. blood urea nitrogen, creatinine, electrolytes) and water, but prevent the loss of blood components and plasma proteins. Two mechanisms act to prevent albumin from being lost from the body by filtration through the glomerular membrane. The first relates to the glomerular membrane pore size, which is small compared to the size and shape of albumin. The second mechanism involves the negatively charged sialo-protein-rich electrical charge on the surface of endothelial cells covering the inner surface of the basement membrane. Since albumin has a net negative charge, it is electrostatically repulsed by the glomerular membrane. As a result, it has been estimated that only about 2 g of albumin was filtered each day across the glomerular capillary wall. Creatinine, on the other hand, lacks electrical charges at physiological pH and hence is able to escape across the muscle cell membrane. It is found that highly alkaline urine occurs in CRF. Our in vitro results could be correlated with this fact i.e. under alkaline conditions interaction of toxins enhances due to increased affinity with HSA. Thus, elimination of toxin would be more feasible at physiological pH.

**Figure 11:**
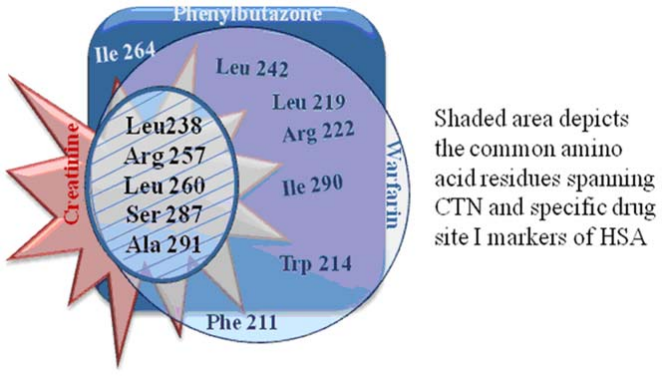
Common amino acid residues between creatinine and HSA. The shaded area depicts the common amino acid residues spanning uremic toxin creatinine and specific drug site I markers of HSA.

Furthermore, the major changes in ASA occur for the residues (role in binding) belonging to the hydrophobic pocket of site I. These residues were overlapping to those of site I markers (Table 4) thereby again supporting the results of site I displacement (Figure 11). Whereas, the residues involved in binding of CTN to site II (Table 5) were non overlapping to those of respective markers, moreover the no. of such residues was less than that for site I.

**Figure 12:**
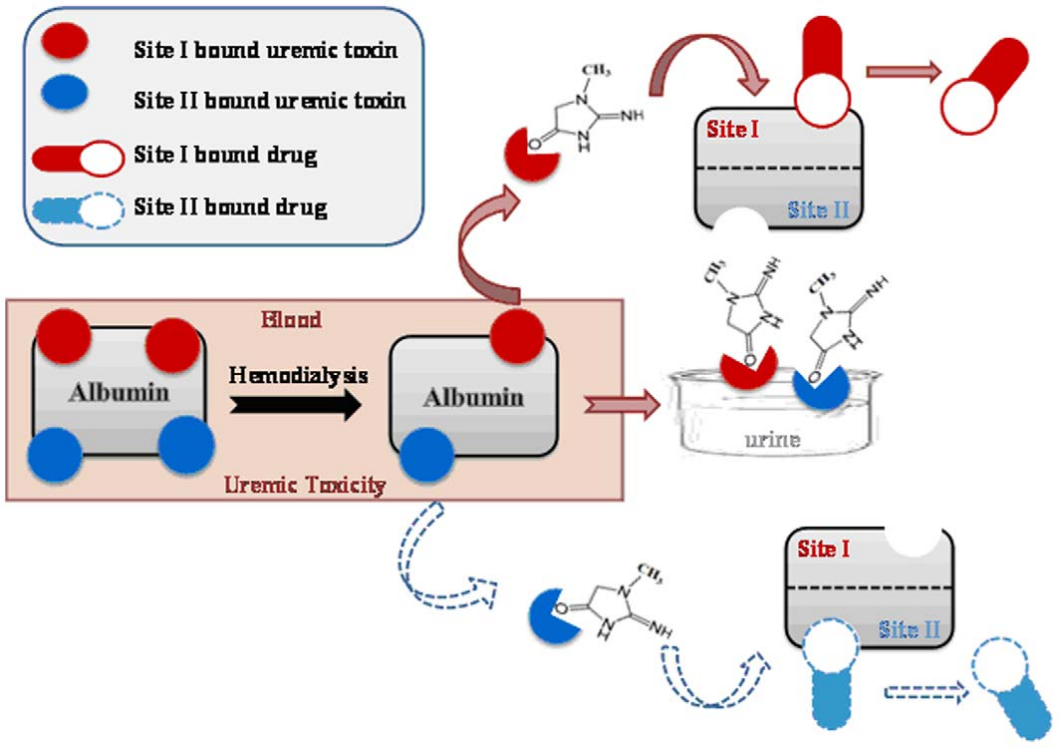
Correlation between uremic toxicity and impaired drug binding. Mechanism showing possible cascade displacement model in uremic toxin– drug system depicts allosteric effect of toxin when binding to site I and site II. Red colour: CTN binding to its high-affinity site, site I as shown by solid arrows; blue colour: CTN binding to its low-affinity site, site II as shown by dashed arrows. Hemodialytic reaction occurring within the blood plasma has been shown in red box.

Curry *et al.* have determined the crystal structure of HSA complexed with five molecules of fatty acid at 2.5 Å resolution [20]. Arg 257 was found to interact with myristate bound to subdomain IIA. Similar interaction we observed with HSA-CTN complexation. It was noteworthy that the single tryptophan residue of HSA (Trp 214) was not in the immediate environment of the docked toxin molecule as proved by our spectroscopic results and was further confirmed by computational mapping approaches. Guanidino compounds are generated *in vivo* as a result of protein and amino acid metabolism. In general the GCs acquire the guanidino group from arginine, with subsequent methylation to creatine and further metabolization to creatinine [41]. In patients with renal impairment, several-fold increases in specific guanidino compounds were observed due to the impaired renal function and altered metabolism [6]. Most drugs are bound to serum proteins to a various degree. Only unbound or free drug is pharmacologically active. There is equilibrium between bound and free drugs, and concentration of free drug can be predicted from total drug concentration. However, under uremic conditions this equilibrium is disturbed and the measured free drug concentration can be significantly higher than expected from total drug concentrations, especially for strongly protein-bound drugs. In such case a patient may experience drug toxicity (Figure 12).

Furthermore, creatinine is removed by hemodialytic strategies [42] but under diseased states uremic compounds of low molecular mass can displace strongly albumin-bound drugs from binding sites responsible for the binding defect of various drugs in uremic sera. To account for the mechanisms that govern such specificity in inhibitory potency, we carefully examined the relationship between the drug binding site(s) and the uremic toxin binding sites. In summary, accumulation of creatinine in patients with renal failure appears to account for a substantial portion of the impaired serum protein binding of drug especially at site I. Consequently, interactions of toxin and drug with respect to serum protein binding and renal excretion may increase the free fraction of drug in serum of patients with renal insufficiency [43]. These findings lend further support to the hypothesis that a retained ligand(s) was responsible for impaired plasma binding associated with uremia and suggests a role for CTN known to accumulate in renal failure.

Renal failure not only alters the renal elimination but also the non renal elimination disposition of drugs that are extensively metabolized by the liver. Clearance of CTN from the blood depends on intramuscular CTN levels, hormone levels, muscle mass, and kidney function/glomerular filtration rate. Creatinine is representative for small uremic toxins, important in clinical analytic domain for it is used as a probe to evidence renal failure or muscular dysfunction. Consequently, accumulation of uremic toxin and slight increase of pH may cause increase of the free fraction of drug due to interaction of toxin in patients with renal failure. Thus, accumulation of uremic toxin in body can be due to conformational change caused by neutral to basic transition which in turn may affect the site–site interactions between domain IIA and domain IIIA such that site interactions between domains will disappear. The findings obtained here will provide useful information for elucidating the complicated mechanism of drug and toxin disposition in renal disease state. This is the current interest of the pharmaceutical companies. This is extremely important for various diseases such as muscular dystrophy and diabetes. Most of the future research and advances well be spun off of these clinical studies.

## Supporting Information

Figure S1 **Rayleigh light scattering of HSA.** The Rayleigh light scattering of HSA at pH 7.0 measured on Hitachi (F-4500) fluorescence spectrophotometer. The excitation and emission wavelengths were set at 350 nm. Both slit widths were set at 10 nm and emission was scanned at time intervals ranging from 0 to 3600 sec (1 hr) by maintaining the temperature at 25°C.
(TIF)
