# Supplementary figures and images for: Elimination of Endogenous Toxin, Creatinine from Blood Plasma Depends on Albumin Conformation: Site Specific Uremic Toxicity & Impaired Drug Binding
Source: PLoS One. 2011 Feb 28;6(2):e17230. doi: 10.1371/journal.pone.0017230 (PMC3046181; doi:10.1371/journal.pone.0017230)

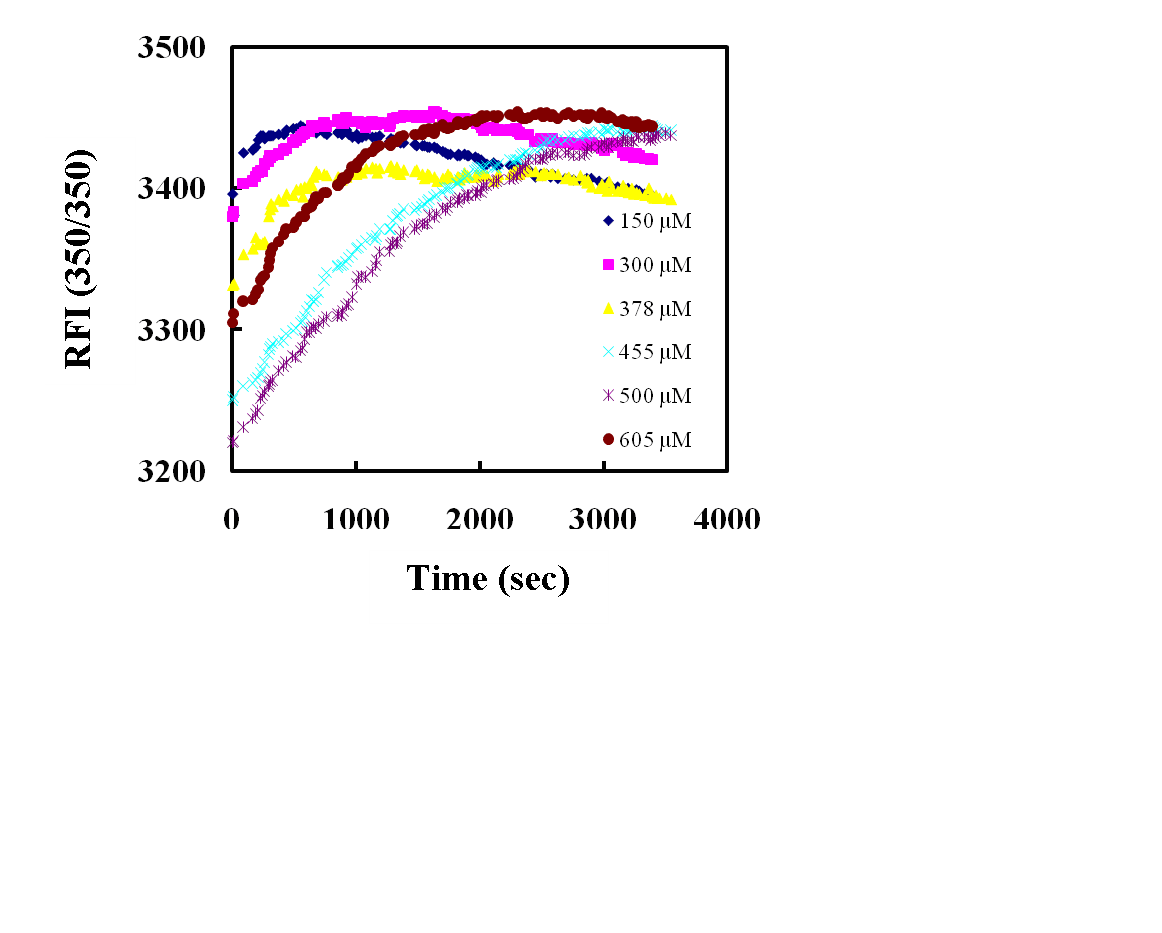

Supplement: Figure S1 — Rayleigh light scattering of HSA. The Rayleigh light scattering of HSA at pH 7.0 measured on Hitachi (F-4500) fluorescence spectrophotometer. The excitation and emission wavelengths were set at 350 nm. Both slit widths were set at 10 nm and emission was scanned at time intervals ranging from 0 to 3600 sec (1 hr) by maintaining the temperature at 25°C. (TIF) [file pone.0017230.s001.tif]
